# Revision of sinistral land snails of the genus *Camaena* (Stylommatophora, Camaenidae) from China based on morphological and molecular data, with description of a new species from Guangxi, China

**DOI:** 10.3897/zookeys.584.7173

**Published:** 2016-04-25

**Authors:** Hong-Li Ding, Pei Wang, Zhou-Xing Qian, Jun-Hong Lin, Wei-Chuan Zhou, Chung-Chi Hwang, Hong-Mu Ai

**Affiliations:** 1 College of Plant Protection, Fujian Agriculture and Forestry University, Fuzhou, Fujian 350002, China; 2 Key Laboratory of Molluscan Quarantine and Identification of AQSIQ, Fujian Entry-Exit Inspection & Quarantine Bureau, Fuzhou, Fujian 350001, China; 3 Zhejiang Museum of Natural History, Hangzhou, Zhejiang, 310014, China; 4 Department of Life Sciences, National University of Kaohsiung, No.700, Kaohsiung University Road, Nan-Tzu District, Kaohsiung 81148, Taiwan

**Keywords:** Land snail, Gastropoda, camaenid, taxonomy, anatomy, molecular phylogeny

## Abstract

The camaenid land snail genus *Camaena* is widely distributed throughout Southeast Asia. Thirteen species are found in China alone. Among these, *Camaena cicatricosa* (Müller, 1774) is the most widely distributed species, including four subspecies, *Camaena cicatricosa ducalis* (Ancey, 1885), *Camaena cicatricosa inflata* (Möllendorff, 1885), *Camaena cicatricosa obtecta* (Fischer, 1898) and *Camaena cicatricosa connectens* (Dautzenberg & Fischer, 1906). The systematics of these taxa is revised herein based on comparative shell morphology and anatomy as well as analyses of DNA sequences of two mitochondrial genes (COI, 16S rRNA) and one nuclear marker, ITS2. We found that all subspecies form well-supported clades in a molecular phylogeny and are well-differentiated from each other by genetic distances that are consistent with amounts of interspecific differentiation. In addition, they clearly differ from each other in reproductive features. Based on these observations, we elevate all four subspecies to the rank of full species. Moreover, based on morphological and mitochdondrial differentiation, we describe a new species, *Camaena poyuensis* sp. n. from Guangxi, China. The new species conspicuously differs from its sibling species *Camaena cicatricosa* in having a larger and more depressed shell, a completely covered umbilicus, more or less purplish peristome, an obtuse angle at the junction of the basal and columellar lip, longer pedunculus of the bursa copulatrix, thicker epiphallus and penis, and short conic verge. Previous named species are also redescribed on their shell and anatomical characters, because the original descriptions are uninformative.

## Introduction

The genus *Camaena* Albers, 1850, with the type species *Helix cicatricosa* Müller, 1774, is distributed throughout Southeast Asia where it occurs in southern China, Indo-China, Eastern India, the Philippines and Sulawesi (Zilch 1959–1960). In China, this genus is found mainly in the provinces of Hainan, Guangdong, Guangxi, Yunnan, Guizhou, Hunan, Fujian and Sichuan, but has not been recorded from north of the Yangtze River. Thirteen species of *Camaena* have been recorded in China (Pilsbry 1894, Yen 1939, Richardson 1985, Chen and Gao 1987, Chen 1990, Chen and Zhang 1999, Schileyko 2003, Wang et al. 2014).

The shells of all but two Chinese species are dextral. Among the two sinistral species, *Camaena seraphinica* Heude, 1890 is known only from the type locality, Dingan Town, Tianlin County (formerly “Si-lin”), Guangxi Province. The type locality of the second sinistral species, *Camaena cicatricosa cicatricosa* (Müller, 1774) is unknown, but specimens exhibiting typical features, such as a sinistral, umbilicated, subcarinated, depressed-globular, yellowish shell with numerous chestnut coloured bands, are widely distributed throughout southern China (Möllendorff 1885, Pilsbry 1891). They can be found in Guangdong, Guangxi, Yunnan, Guizhou, Hunan and Hong Kong (Yen 1939, Chen and Gao 1987, Zhang 2008, Wang et al. 2014).

So far, anatomical characters of sinistral camaenids have not been studied except for *Camaena cicatricosa* (Schileyko 2003). The shell morphology of *Camaena cicatricosa* is variable. Four infraspecific names have been proposed, these being *Camaena cicatricosa ducalis* (Ancey, 1885) and *Camaena cicatricosa inflata* (Möllendorff, 1885) from Guizhou, China, *Camaena cicatricosa obtecta* (Fischer, 1898), and *Camaena cicatricosa connectens* (Dautzenberg & Fischer, 1906) from northern Vietnam. These subspecies differ from the nominate form in shell features, such as shell dimensions and shape, openness of umbilicus, sharpness of peripheral angle, convexity of whorls and the presence of a hump beside umbilicus. *Camaena cicatricosa ducalis* is distinguished from other subspecies by its much larger and stronger malleated shell, a more dilated columellar margin and an almost completely covered umbilicus (Ancey 1885). *Camaena cicatricosa inflata* differs from the nominate form in having a much more globular shell, with obsolete peripheral angle and more inflated and gibbous last whorl and nearly closed umbilicus (Möllendorff 1885). *Camaena cicatricosa obtecta* is characterized by having a more globular shell, with weak peripheral angle, a completely closed umbilicus and a nearby umbilical hump (Fischer 1898). *Camaena cicatricosa connectens* differs from *Camaena cicatricosa cicatricosa* by having fine and tight granules on shell (Dautzenberg and Fischer, 1906). These taxa have previously been treated either as synonyms, varieties or subspecies of *Camaena cicatricosa* by different authors based on comparative shell morphology (Pilsbry 1891, 1894, Fischer and Dautzenberg 1904, Dautzenberg and Fischer 1906, Yen 1939, Zilch 1964, Chen and Gao 1987, Schileyko 2011) without reaching a consensus.

Owing to the incongruent delimitation of species in the past, which reflect exclusive reliance on shell characters and the incomplete description of these species, an up-dated revision using modern techniques and species delimitation is required. The mtDNA COI (cytochrome c oxidase subunit I) gene is a commonly used marker for the DNA barcode identification system and is potentially useful for species discovery and identification (Hebert et al. 2003). Additional genetic markers on mitochondrial and nuclear genome have been used alongside COI fragment (e.g., Wu et al. 2008, Criscione and Köhler 2014). The present study aims to resolve the phylogenetic relationships of *Camaena cicatricosa*, to correctly delimit its closely related allies and to describe a putatively new species based on comparative analyses of morphological and molecular characters.

## Material and methods

**Figure 1.**
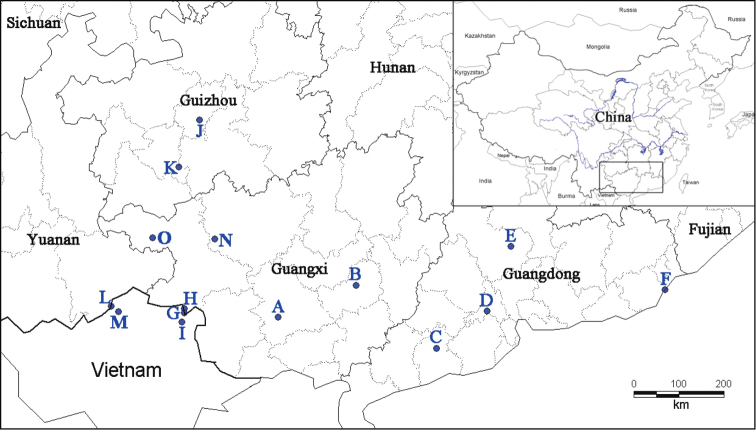
Map of locations of *Camaena* species. *Camaena cicatricosa*: **A** Nanning, Guangxi, China **B** Guiping, Guangxi, China **C** Yangchun, Guangdong, China **D** Gaoming, Canton, Guangdong, China **E** Yingde, Guangdong, China **F** Shantou, Guangdong, China. *Camaena obtecta*: **G** Buhaitun, Jinxi, Guangxi, China **H** Longbang, Jingxi, Guangxi, China **I** Cao Bang, Vietnam (type locality). *Camaena inflata*: **J** Qianlin park, Guiyang, Guizhou, China **K** Ziyun, Guiyang, Guizhou, China. *Camaena connectens*: **L** Tianbao, Malipo, Yunnan, China **M** Ha Giang, Vietnam (type locality). *Camaena poyuensis* sp. n.: **N** Poyue, Bama, Hechi, Guangxi, China (type locality). *Camaena seraphinica*: **O** Dingan, Tianlin, Guangxi, China (type locality).

This study is based on material collected by the authors at several sites in China (Fig. 1). Live adults were drowned in water for 12–24 hours, then boiled briefly in hot water to ensure their death. Soft body was preserved in 95% ethanol and stored at -40 °C. Empty shells were cleaned and preserved at room temperature. Samples have been deposited in the State Key Laboratory of Molluscan Quarantine and Identification, FJIQBC. Shells were measured to 0.01 mm using electronic calipers. Standard shell parameters were measured on 10–74 specimens per species following Dillon (1984). Genitalia of adult snails were dissected under a dissecting microscope (ZEISS Stemi 2000). The terminology used for the reproductive system follows Gómez (2001). More than three specimens of each species have been dissected.

A piece of foot muscle tissue of about 0.05 g was used for DNA extraction. The muscle tissue was bathed in sterile water for 3–6 hours to remove residual alcohol. Genomic DNA was isolated using a DNeasy Blood and Tissue Kit (Qiagen, Beijing), examined by agarose gel electrophoresis, and stored at -20 °C for further use. Three specimens from each sampling locality were used for DNA extraction. Fragments of the partial mitochondrial cytochrome c oxidase subunit 1 (COI) and 16S rRNA (16S), and the internal transcribed spacer 2 (ITS2) region of nuclear ribosomal DNA were amplified by PCR using the primer pairs and amplification conditions listed in Table 1.

**Table 1.** Primer pairs and PCR conditions used in the analysis of the COI, 16S rRNA and ITS2 genes of *Camaena*.

Gene	Primer pairs (5’-3’)	Cycling conditions	Reference
COI	LCO:GGTCAACAAATCATAAAGATATTGG HCO:TAAACTTCAGGGTGACCAAAAAATCA	94°: 30s; 94°: 10s, 45°: 50s, 72°: 1min, 40 cycles; 72°: 10min.	Folmer et al. 1994
16S	16SAR: CGCCTGTTTATCAAAAACAT 16SBR: CCGGTCTGAACTCAGATCACGT	94°: 30s; 94°: 10s, 45°: 50s, 72°: 1min50s, 40 cycles; 72°: 10min.	Palumbi et al. 1991
ITS2	FYIT2:CATCGACATCTTGAACGCACAT RYIT2: TCCCAAACAACCCGACTCCT	94°: 30s; 94°: 10s, 55°: 30s, 72°: 1min30s, 40 cycles; 72°: 10min.	Present study

Both strands of PCR products were purified and sequenced by use of the PCR primers. After sequencing, raw sequence files were proof-read based on chromatograms and assembled in BioEdit 7.2 (Hall 1999). ITS2 sequences were annotated by using HMMer (Eddy 1998) and ITS2 Database (Koetschan et al. 2010). Sequence alignments were generated using ClustalW implemented in MEGA6 (Tamura et al. 2013). In the 16S alignment, sequences of ambiguous alignment were removed using Gblocks v. 0.91b (Castresana 2000), with the minimum number of sequences for a conserved position set to 22, the minimum number of sequences for a ﬂanking position set to 36, the maximum number of contiguous non-conserved positions set to 8, the minimum length of a block set to 4, and no gap positions are allowed. Sequences were checked for saturation using the test implemented in DAMBE 5.3 (Xia et al. 2003, Xia and Lemey 2009, Xia 2013). Pairwise *p*-distances between taxa were calculated using MEGA6 under the option pairwise deletion of gaps. Prior to the model-based phylogenetic analyses, the best-ﬁtted model of nucleotide substitution was determined for each gene separately using the Akaike Information Criterion calculated with jModelTest v2.1.7 (Darriba et al. 2012, Guindon and Gascuel 2003). Sequences of the three genes were then concatenated into one partitioned data set. Unique sequences were identified using DAMBE 5.3. Maximum likelihood (ML) analyses were conducted using RaxML v8.2.4 (Stamatakis 2014) by applying the GTRGAMMAI model, with parameters estimated from the data, to separate partitions for each gene. The branch support of the ML tree was estimated by using the bootstrapping criteria autoMRE (Majority Rule Criterion) implemented in RAxML. *Bradybaena sequiniana* (Heude, 1885) (Bradybaenidae) and *Cornu aspersum* (Müller, 1774) (Helicidae) were used as outgroup. Two additional dextral *Camaena* species were also analyzed for comparisons.

### Abbreviations

16S, 16S rRNA gene; COI, cytochrome c oxidase subunit 1 gene; FJIQBC, Fujian Entry-Exit Inspection & Quarantine Bureau, Fuzhou, Fujian, China; ITS2, internal transcribed spacer 2 region of nuclear ribosomal DNA; IZCAS, Institute of Zoology, Chinese Academy of Science Museum, Beijing, China; ML, Maximum Likelihood; MNHN, Muséum National d’Histoire Naturelle, Paris, France; SMF, Naturmuseum Senckenberg, Frankfurt/Main, Germany.

## Results

### Molecular analysis

Molecular phylogenetic analyses were based on DNA sequences from forty-one specimens of *Camaena* from 14 localities as well as sequences from two additional specimens of *Bradybaena sequiniana* and *Cornu aspersum* that were used as outgroup to root the tree (Table 2). Hence, a total of 129 sequences were newly generated and deposited in GenBank (Table 2). The final sequence alignments had lengths of 601 bp (COI), 348 bp (16S) and 586 bp (ITS2), respectively. Poorly aligned segments of the 16S alignments were removed using Gblocks. Gblocks maintained 348 conserved alignment positions in 16S. For phylogenetic analyses, the three sequence data sets were concatenated into one, with a length of 1,535 bp. The concatenated alignment contained 29 unique sequences, which were used for subsequent analyses. Xia’s et al. (2003) test indicated no or little saturation in the three fragments, with Iss.c values significantly larger than Iss values (p < 0.01). Separate models determined by jModeltest for the three genes revealed the GTR model with gamma distribution and proportions of invariable sites (GTR+G+I) as the best-fit substitution model for COI, TPM2uf with gamma distribution (TPM2uf+G) for 16S, and HKY model with gamma distribution (HKY+G) for ITS2. Hence, the most complex model, GTR+G+I, among the three selected models was used for the maximum likelihood analysis.

**Table 2.** Sampling information and GenBank accession numbers. Localities are all in China otherwise noted.

Species / Locality	Coordinates	Collection date	COI	16S	ITS2
***Camaena cicatricosa***					
Guiping, Guangxi	23°23'58"N; 110°03'44"E	2013.11.02	KU061276 KU061277 KU586516	KU586474 KU586475 KU586476	KU586555 KU586556 KU586557
Yingde, Guangdong	24°09'44"N; 113°24'06"E	2014.09.17	KU586533 KU586534 KU586535	KU586495 KU586496 KU586497	KU586576 KU586577 KU586578
Gaoming, Guangdong	22°54'8"N; 112°53'2"E	2009.10.22	KU586513 KU586514 KU586515	KU586471 KU586472 KU586473	KU586552 KU586553 KU586554
Nanning, Guangxi	22°47'27"N; 108°23'33"E	2013.05.18	KU586521 KU586522 KU586523	KU586483 KU586484 KU586485	KU586564 KU586565 KU586566
Yangchun, Guangdong	22°10'3"N; 111°47'8"E	2014.04.01	KU586530 KU586531 KU586532	KU586492 KU586493 KU586494	KU586573 KU586574 KU586575
Shantou, Guangdong	23°16'60"N; 116°44'23"E	2010.11.03	KU586527 KU586528 KU586529	KU586489 KU586490 KU586491	KU586570 KU586571 KU586572
***Camaena obtecta***					
Longbang, Guangxi	22°53'00"N; 106°19'34"E	2015.10.03	KU055610 KU055611 KU586517	KU586477 KU586478 KU586479	KU586558 KU586559 KU586560
Buhaitun, Guangxi	22°52'38"N; 106°19'39"E	2015.10.03	KU586508 KU586509 KU586510	KU586465 KU586466 KU586467	KU586546 KU586547 KU586548
***Camaena inflata***					
Guiyang, Guizhou	26°36'8"N; 106°41'15"E	2008.10.16	KU586524 KU586525 KU586526	KU586486 KU586487 KU586488	KU586567 KU586568 KU586569
Ziyun, Guizhou	25°41'42"N; 106°14'31"E	2014.04.18	KU586536 KU586537 KU586538	KU586498 KU586499 KU586500	KU586579 KU586580 KU586581
***Camaena connectens***					
Tianbao, Malipo, yunnan	22°57'57"N; 104°49'25"E	2015.04.17	KU586518 KU586519 KU586520	KU586480 KU586481 KU586482	KU586561 KU586562 KU586563
***Camaena poyuensis* sp. n.**					
Poyue town, Bama, Hechi, Guangxi	24°17'30"N; 107°05'32"E	2014.05.25	KU061273 KU586511 KU586512	KU586468 KU586469 KU586470	KU586549 KU586550 KU586551
***Camaena menglunensis***					
Xishuangbanna, Yunnan	21°51'36"N; 101°24'53"E	2011.07.24	KU586506 KU586507	KU586463 KU586464	KU586544 KU586545
***Camaena jinpingensis***					
Jinping, Yunnan	22°53'18"N; 103°20'23"E	2011.07.29	KU586503 KU586504 KU586505	KU586460 KU586461 KU586462	KU586541 KU586542 KU586543
***Bradybaena sequiniana* (Family Bradybaenidae)**					
Badong, Hubei	31°02'46"N; 110°22'18"E	2011.06.13	KU586501	KU586458	KU586539
***Cornu aspersum* (Family Helicidae)**					
Italy		2010.04.06	KU586502	KU586459	KU586540

**Figure 2.**
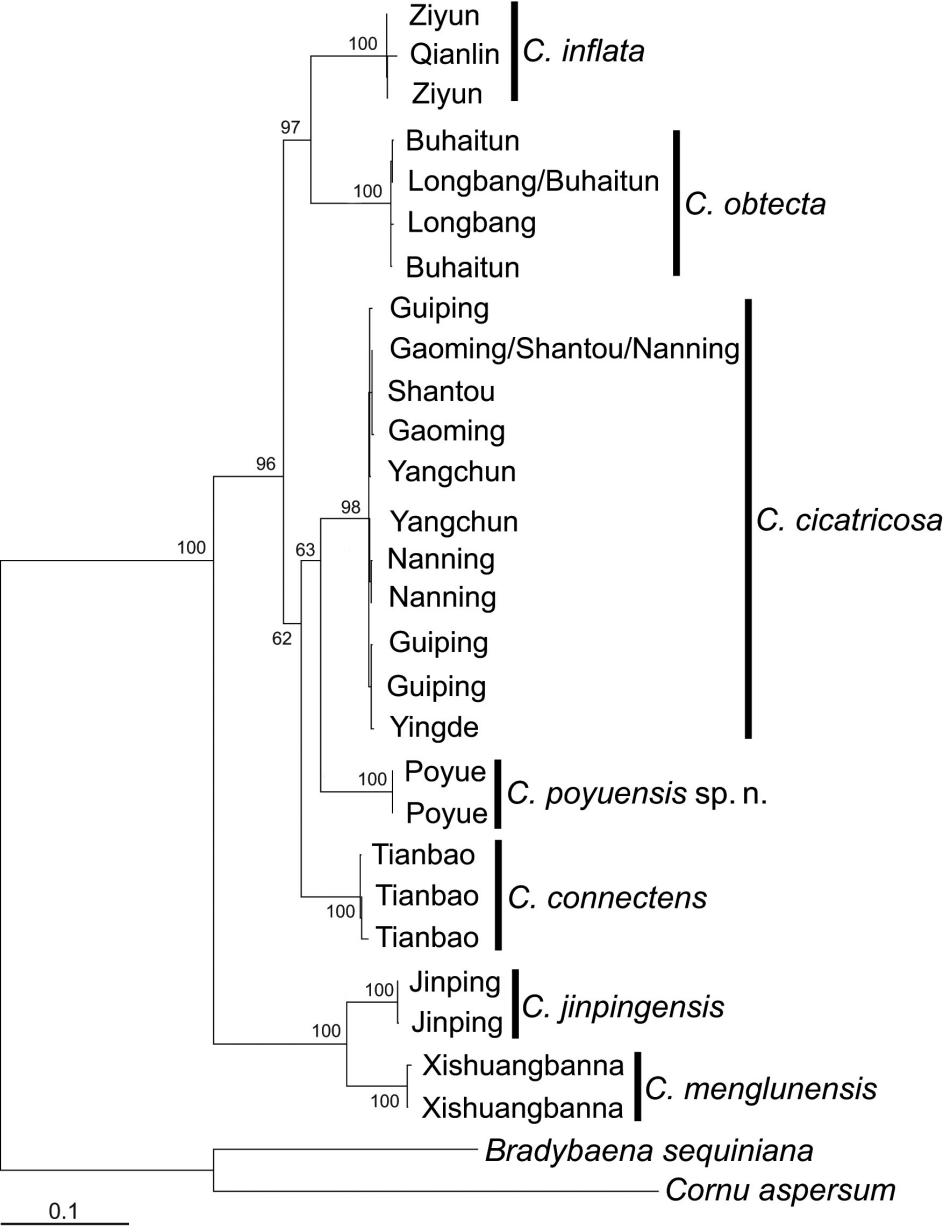
Maximum Likelihood tree based on analysis of the concatenated dataset of COI, 16S and ITS2 sequences. Numbers beside nodes indicate bootstrapping support (%) for main clades. See Table 2 for sampling locations.

The bootstrap support values smaller than 50% were considered poorly supported and are not considered below. Seven *Camaena* clades, which represent candidate species, with terminal clustering of sequences were identified from the reconstructed phylogeny. Although only a limited number of species was sampled, the phylogeny confirmed the monophyly of the five sinistral species from China (Fig. 2).

Genetic distances between the seven *Camaena* clades varied from 13 to 22% (average = 17%) in COI, from 5 to 15% (average = 12%) in 16S, and from 1 to 12% (average = 7%) in ITS2 (Table 3). Within-clade genetic distances were lower than 1.1% (average = 0.4%) in COI, 0.4% (average = 0.1%) in 16S, and 1.3% (average = 0.3%) in ITS2. Genetic distance between the six populations of *Camaena cicatricosa cicatricosa* ranged from 0 to 1.5% (average = 0.9%) in COI, 0% in 16S, and 0 to 0.4% (average = 0.2%) in ITS2. The genetic distance showed no overlap between within-clade and between-clade, with an exception of ITS2 which had slight overlap.

**Table 3.** Average genetic distance of COI, 16S and ITS2 genes between and within (diagonal) species.

**COI**		1	2	3	4	5	6	7	8	9
1	*Camaena cicatricosa*	0.008								
2	*Camaena inflata*	0.166	0.011							
3	*Camaena obtecta*	0.164	0.180	0.003						
4	*Camaena connectens*	0.133	0.184	0.165	0.000					
5	*Camaena poyuensis* sp. n.	0.135	0.178	0.169	0.126	0.000				
6	*Camaena jinpingensis*	0.178	0.200	0.189	0.179	0.193	0.001			
7	*Camaena menglunensis*	0.183	0.200	0.217	0.181	0.181	0.133	0.003		
8	*Bradybaena sequiniana*	0.214	0.229	0.220	0.215	0.220	0.230	0.233	-	
9	*Cornu aspersum*	0.215	0.220	0.237	0.228	0.223	0.233	0.235	0.240	-
**16S**		1	2	3	4	5	6	7	8	9
1	*Camaena cicatricosa*	0.000								
2	*Camaena inflata*	0.083	0.003							
3	*Camaena obtecta*	0.099	0.100	0.004						
4	*Camaena connectens*	0.072	0.101	0.141	0.000					
5	*Camaena poyuensis* sp. n.	0.052	0.106	0.114	0.101	0.000				
6	*Camaena jinpingensis*	0.121	0.138	0.147	0.132	0.144	0.000			
7	*Camaena menglunensis*	0.129	0.149	0.143	0.132	0.152	0.066	0.000		
8	*Bradybaena sequiniana*	0.210	0.227	0.216	0.224	0.230	0.218	0.221	-	
9	*Cornu aspersum*	0.230	0.244	0.237	0.244	0.236	0.241	0.239	0.247	-
**ITS2**		1	2	3	4	5	6	7	8	9
1	*Camaena cicatricosa*	0.002								
2	*Camaena inflata*	0.016	0.000							
3	*Camaena obtecta*	0.028	0.012	0.001						
4	*Camaena connectens*	0.034	0.019	0.012	0.013					
5	*Camaena poyuensis* sp. n.	0.028	0.024	0.027	0.032	0.000				
6	*Camaena jinpingensis*	0.114	0.113	0.118	0.122	0.104	0.000			
7	*Camaena menglunensis*	0.116	0.114	0.120	0.123	0.103	0.014	0.006		
8	*Bradybaena sequiniana*	0.245	0.242	0.249	0.250	0.243	0.244	0.238	-	
9	*Cornu aspersum*	0.291	0.287	0.288	0.291	0.302	0.292	0.285	0.265	-
										

Abbreviations: B., Bradybaena; Co., Cornu.

The well supported clades by means of bootstrap values and sufficient differentiation between clades in terms of branch lengths and genetic distances well delimited these clades as species rank. Six of the seven clades were recognized as already described species or subspecies and one clade represented a new taxon, after examining morphological characters (see Systematics part below). The comparative anatomy and nomenclatural act will be assessed in Systematics part. For clarifying, these recognized species have been labeled in figures and tables with the names and ranks of taxa treated or described below.

## Systematics

### Camaenidae Pilsbry, 1895

#### Camaena

Taxon classificationAnimaliaStylommatophoraCamaenidae

Albers, 1850

##### Type species.

*Helix cicatricosa* Müller, 1774, subsequent designation by Martens, 1860.

#### Camaena cicatricosa

Taxon classificationAnimaliaStylommatophoraCamaenidae

(Müller, 1774)

**Figure 3.**
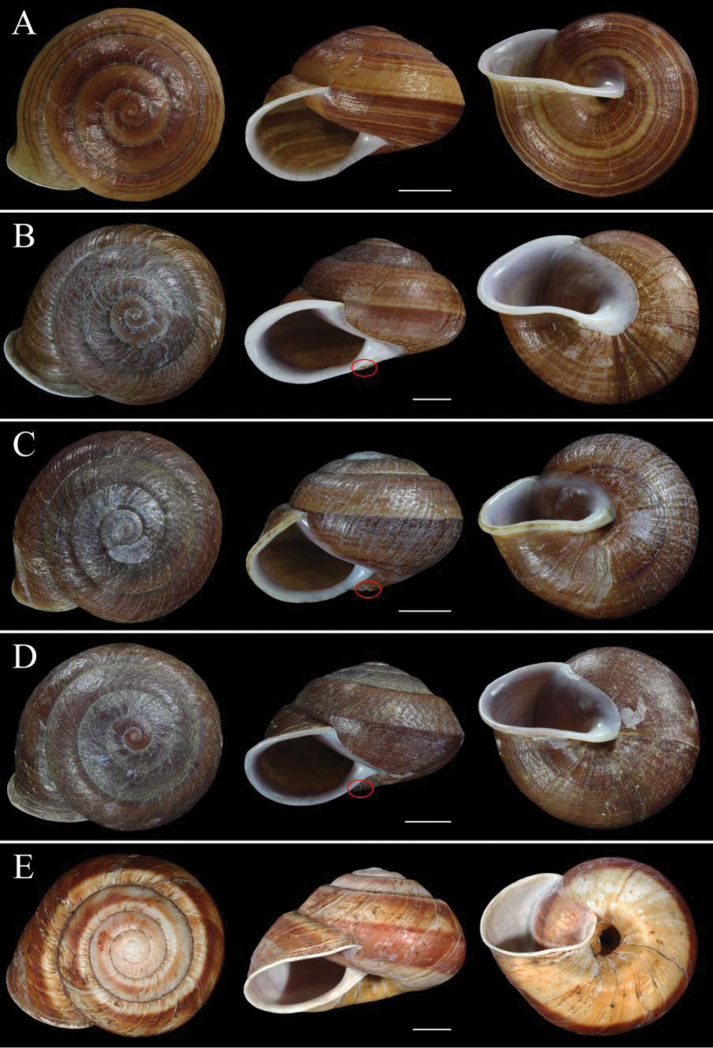
Shells of sinistral Chinese species of *Camaena*. **A** *Camaena cicatricosa* (FJIQBC 18483, Guiping, Guangxi, China) **B** *Camaena obtecta* (FJIQBC 18743, Longbang, Jingxi, Guangxi, China) **C** *Camaena inflata* (FJIQBC 18782, Qianlin park, Guiyang, Guizhou, China) **D** *Camaena connectens* (FJIQBC 18826, Tianbao, Malipo, Yunnan, China) **E** *Camaena seraphinica* (syntype, IZCAS HMT-0001a; Dingan, Tianlin, Guangxi, China). Red circle indicates a hump beside the umbilicus. Scale = 10 mm.

**Figure 4.**
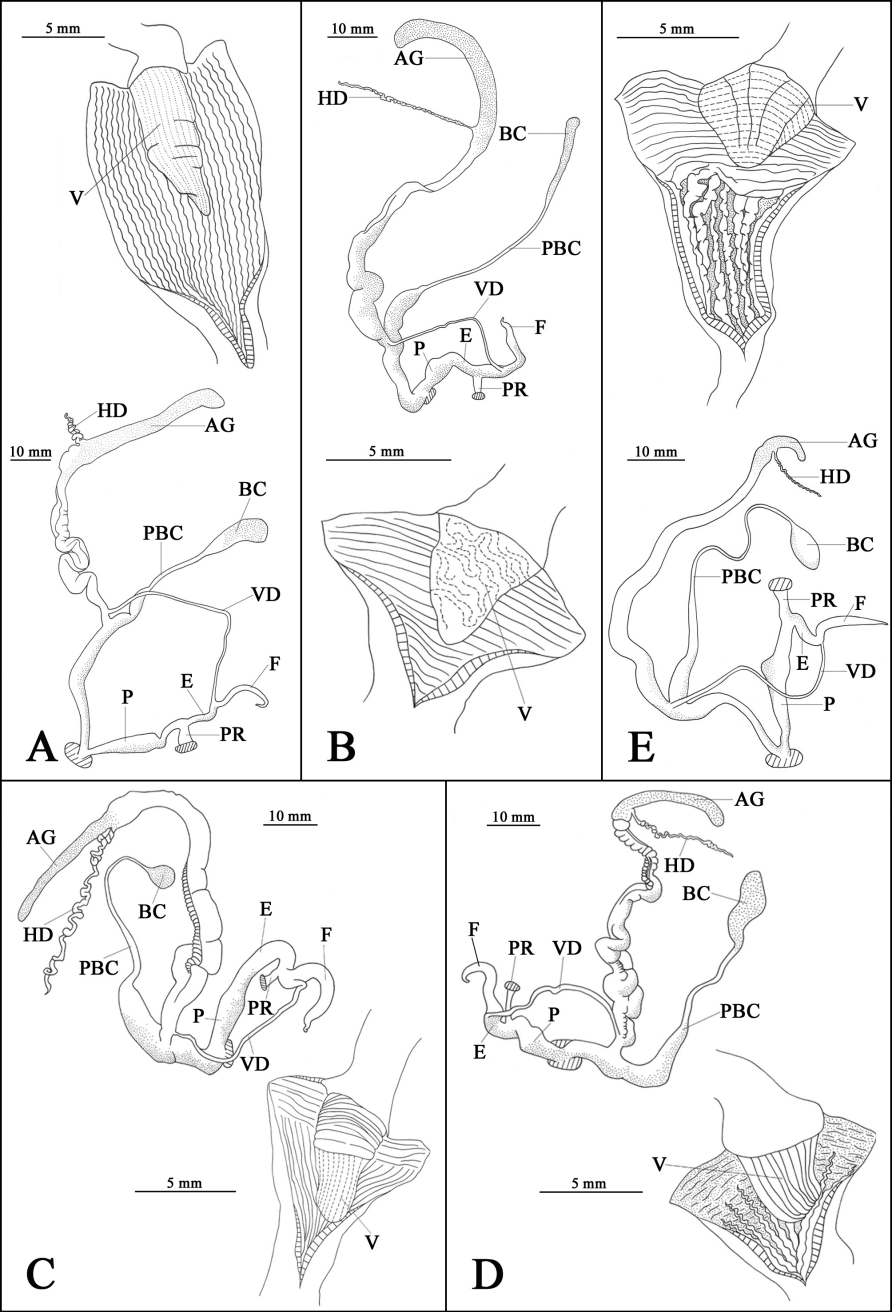
Reproductive system of sinistral Chinese sinistral species of *Camaena*. **A** *Camaena cicatricosa* (FJIQBC 18483, Guiping, Guangxi, China) **B** *Camaena obtecta* (FJIQBC 18743, Longbang, Jinxi, Guangxi, China) **C** *Camaena inflata* (FJIQBC 18797, Qianlin park, Guiyang, Guizhou, China) **D** *Camaena connectens* (FJIQBC 18832, Tianbao, Malipo, Yunnan, China) **E** *Camaena poyuensis* sp. n. (FJIQBC 18484, Poyue, Bama, Guangxi, China). Abbreviations: V, verge; AG, albumen gland; BC, bursa copulatrix; E, epiphallus; F, flagellum; HD, hermaphroditic duct; P, penis; PR, penis retractor muscle; PBC, pedunculus of bursa copulatrix; VD, vas deferens.

Figs 3A , 4A , Table 4

Helix cicatricosa Müller, 1774: 42; Chemnitz 1786: 90–91, pl. 109, fig. 923; Albers 1850: 85; Férussac and Deshayes 1850: 168–169, pl. 41, fig. 1–2; Martens 1867: 47.
Nanina (Ariophanta) cicatricosa , Beck 1837: 5.
Helix (Camaena) cicatrosa , Adams and Adams 1855: 189 [sic.]
Helix (Camaena) cicatricosa , Pilsbry 1891: 198, pl. 21, fig. 45–47; Fischer 1898: 314.
Camaena (Camaena) cicatricosa , Pilsbry 1894: 103, pl. 19, fig. 8; Zilch 1964: 243, 1960 [in 1959–1960]: 606, fig. 2125.
Camaena cicatricosa , Fischer and Dautzenberg 1904: 399; Dautzenberg and Fischer 1906: 353, 355; Yen 1939: 123, pl. 12, fig. 32; Solem 1992: 7–8, figs 1–3; Chen and Gao 1987: 100–101, fig. 129; Schileyko 2003: 1511, fig. 1947, 2011: 41; Hwang 2011: 5, fig. 2; Wang et al. 2014; Qian and Zhou 2014: 123.

##### Type locality.

Unknown.

##### Material examined.

See Table 4.

**Table 4.** Measurements of shells (mm). Localities are all in China otherwise noted. **n** sample size **SH** shell height **SW** shell width **AH** aperture height **AW** aperture width.

Species / Locality	Voucher	SH	SW	SW/SH	AH	AW	AW/AH
***Camaena cicatricosa***							
Guiping, Guangxi	FJIQBC 18503–18542 (n = 40)	21.26–32.86	35.00–42.64	1.28–1.65	16.68–21.44	21.26–27.40	1.18–1.34
		(27.61±3.17)	(39.50±2.21)	(1.44±0.11)	(19.53±1.33)	(24.36±1.74)	(1.25±0.04)
Yingde, Guangdong	FJIQBC 18543–18616 (n = 74)	26.78–36.00	39.10–48.74	1.35–1.56	19.62–24.92	22.10–22.90	1.05–1.26
		(28.66±2.45)	(41.87±2.83)	(1.46±0.05)	(21.28±0.05)	(24.94±2.30)	(1.17±0.06)
Gaoming, Guangdong	FJIQBC 18617–18640 (n = 24)	26.48–33.30	38.20–44.84	1.31–1.56	19.20–21.56	24.64–29.60	1.23–1.37
		(30.53±2.45)	(41.40±2.19)	(1.43±0.22)	(20.61±0.09)	(27.1±0.17)	(1.32±0.05)
Nanning, Guangxi	FJIQBC 18641–18670 (n = 30)	22.10–30.00	36.26–43.76	1.43–1.64	18.08–21.88	22.00–18.64	1.19–1.32
		(26.59±2.12)	(40.95±2.24)	(1.54±0.07)	(20.31±1.13)	(25.48±1.73)	(1.26±0.04)
Yangchun, Guangdong	FJIQBC 18671–18710 (n = 40)	23.44–30.00	33.76–44.26	1.20–1.52	15.86–21.66	19.42–27.06	1.11–1.25
		(27.00±1.94)	(37.76±3.10)	(1.40±0.10)	(18.45±1.61)	(22.07±1.96)	(1.20±0.04)
Shantou, Guangdong	FJIQBC 18711–18742 (n = 32)	23.40–27.26	35.44–39.64	1.44–1.57	17.64–19.36	21.18–24.14	1.19–1.29
		(25.07±1.44)	(37.58±1.53)	(1.50±0.05)	(18.61±0.61)	(22.89±1.04)	(1.23±0.04)
***Camaena obtecta***							
Longbang, Guangxi	FJIQBC 18743–18764 (n = 22)	35.76–44.10	53.20–61.74	1.33–1.57	26.70–32.26	33.28–38.80	1.15–1.29
		(39.46±2.22)	(57.93±2.53)	(1.47±0.07)	(29.64±1.70)	(35.54±1.51)	(1.19±0.04)
Buhaitun, Guangxi	FJIQBC 18765–18781 (n = 17)	32.56–40.70	51.64–59.86	1.40–1.59	23.74–30.08	31.54–38.90	1.27–1.37
		(36.90±2.72)	(55.88±2.77)	(1.52±0.06)	(27.12±1.73)	(36.10±2.48)	(1.33±0.03)
***Camaena inflata***							
Guiyang, Guizhou	FJIQBC 18782–18813 (n = 32)	25.90–38.60	38.40–46.08	1.19–1.55	20.28–23.02	22.00–38.24	1.09–1.66
		(29.97±3.31)	(42.87±2.18)	(1.44±0.10)	(21.39±0.80)	(26.50±0.40)	(1.24±0.14)
Ziyun, Guizhou	FJIQBC 18814–18825 (n = 12)	31.66–40.40	48.52–56.66	1.40–1.55	21.74–28.62	27.50–33.20	1.11–1.32
		(35.43±3.08)	(52.15±2.96)	(1.48±0.06)	(25.08±2.43)	(30.43±1.88)	(1.22±0.70)
***Camaena connectens***							
Tianbao, Malipo, yunnan	FJIQBC 18826–18835 (n = 10)	30.40–37.44	48.18–55.26	1.37–1.59	22.08–27.80	29.20–34.10	1.23–1.32
		(34.47±2.69)	(51.61±2.35)	(1.50±0.08)	(24.37±1.86)	(31.14±1.65)	(1.28±0.40)
***Camaena poyuensis* sp. n.**							
Poyue town, Bama, Hechi, Guangxi	Holotype FJIQBC 18484	38.08	56.08	1.47	27.92	34.72	1.24
	Paratypes FJIQBC 18485–18486, 18489–18502 (n = 16)	34.12–41.00	52.50–58.74	1.43–1.63	23.32–29.00	32.00–37.06	1.21–1.38
		(37.02±2.22)	(55.82±1.74)	(1.51±0.06)	(27.09±1.65)	(34.88±1.32)	(1.29±0.05)

note: Coordinates and collection date see Table 2.

##### Diagnosis.

Shell sinistral, medium sized, thick, depressed-globular, yellowish brown, with obtuse apex and high dome-shaped spire; with 5 1/2 rapidly increasing and rather flat whorls separated by deep suture; body whorl convex, not descending behind the aperture; periphery bluntly angulate, becoming round behind aperture. Sculpture of fine, dense, irregular and oblique wrinkles and malleation, with low radiate folds below suture. Aperture roundly lunate, white inside, with curved margin. Peristome white, expanded, slightly reflected, thickened and glossy; columellar margin expanded; inner lip thin, callous. Basal lip curved, forming an obtuse angle at junction with straight and oblique columellar lip. Umbilicus half covered by reflected columellar lip. Color pattern of numerous wavy, reddish brown spiral bands of various thickness; subperipheral and subsutural bands much wider (Fig. 3A)

Penis swollen, tapering distally, with a rounded bulge in correspondence of verge. Epiphallus thin with short, thin and wide penis retractor muscle. Flagellum slender, tapering distally. Vas deferens long and thin. Vagina long and thin, thickened proximally. Bursa copulatrix oval with medium-lengthened and thin pedunculus, expanded at base. Verge long, conic, with dense, weak longitudinal grooves. Inner penial wall supporting longitudinal, prominent and narrowly spaced pilasters (Fig. 4A).

##### Distribution.

Guangdong Province to Nanning, Guangxi (Fig. 1).

##### Ecology.

This species is locally found in high densities in a variety of habitats, which include virgin forests, semi-natural woodland, farmlands and even urban parks. Animals breed during April to September, and reach sexual maturity at 7–8 months of age (Xiao 1989).

##### Comparative remarks.

Distinguished from all the other sinistral species of *Camaena* by its smaller size, having half opened umbilicus, and thin shell, as well as by having only longitudinal pilasters on the inner penial wall, and a long conical penial verge with longitudinal wrinkles. The shell is similar in size as *Camaena inflata*, but the latter differs by having a more globular shape and thicker shell, more convex whorls, and narrowly-spaced transverse wrinkles on the inner penial wall and penial verge.

#### Camaena obtecta

Taxon classificationAnimaliaStylommatophoraCamaenidae

(Fischer, 1898)

Figs 3B , 4B , Table 4

Helix (Camaena) cicatricosa var. obtecta Fischer, 1898: 315, pl. 17, figs 5–6.

##### Type locality.

Vietnam: Luc Chu and Cao Bang

##### Material examined.

See Table 4.

##### Diagnosis.

Shell sinistral, large, thick, solid, depressed-globular, yellowish brown to dark brown, with obtuse apex and low dome-shaped spire; 5 1/2 rapidly increasing and slightly convex whorls separated by deep suture; body whorl expanded, descending in front; periphery bluntly angulate in front of aperture, becoming round behind peristome. Surface with thick growth lines, fine spiral ribs, and weak malleation. Aperture ovate-lunate, white inside, with curved margin. Peristome white, expanded, reflected, thickened and glossy; columellar margin strongly expanded; inner lip thick, callous. Basal lip straight, forming an obtuse angle at junction with straight and oblique columellar lip. Umbilicus completely covered by reflected columellar lip and thickened callus when fully matured. Hump beside umbilicus present. Color pattern of numerous wavy, reddish brown spiral bands of various thickness; subperipheral and subsutural bands much wider (Fig. 3B).

Penis short, swollen, with a rounded bulge in correspondence of verge. Epiphallus medium with short, thin and wide penis retractor muscle. Flagellum elongated, tapering distally. Vas deferens long and thin. Vagina short and thickened. Bursa copulatrix clavate with long and thin pedunculus, apparently expanded at basal one-third its length. Verge conic, with irregular and curly wrinkles. Inner penial wall supporting transverse and narrowly spaced pilasters (Fig. 4B).

##### Distribution.

This species has previously been recorded from Cao Bang and Luc Chu in northern Vietnam. Luc Chu is the area north of Cao Bang to the border with China according to Billet (1898). In addition, it is now recorded from Longbang and Buhaitun, in Jinxi, southwestern Guangxi, China, approximately 20 km north of Cao Bang (Fig. 1).

##### Ecology.

This species inhabits forests on limestone, including degraded forests.

##### Comparative remarks.

This species is characterized in having a hump beside the completely covered umbilicus, thick shell, ovate-lunate aperture, transverse only pilasters on inner penial wall, and a conic verge with irregularly curly wrinkles. It differs from *Camaena cicatricosa* by having a larger shell, a completely covered umbilicus, humped base beside umbilicus, more convex whorls and ovate-lunate aperture. It forms a well-differentiated clade in the phylogenetic tree (Fig. 2) and exhibits sufficient morphological differences to justify elevation to full species rank.

#### Camaena inflata

Taxon classificationAnimaliaStylommatophoraCamaenidae

(Möllendorff, 1885)

Figs 3C , 4C , Table 4

Helix cicatricosa var. inflata Möllendorff, 1885: 393.
Helix (Camaena) cicatricosa var. inflata, Pilsbry 1891: 199, pl. 25, fig. 101.
Camaena cicatricosa var. inflata, Fischer and Dautzenberg 1904: 399; Dautzenberg and Fischer 1906: 355–356; Dautzenberg and Fischer 1908: 172.
Camaena cicatricosa cicatricosa , Yen 1939: 123, pl 12, fig. 33; Zilch 1964: 243; Schileyko 2011: 41.

##### Type locality.

Tshien-ti-shan, province of Guidshou [Tshien-te-shan (Yen, 1939)].

##### Material examined.

Holotype. SMF 8092. Paratype. SMF 8093, 26502.

Additional material see Table 4.

##### Diagnosis.

Shell sinistral, medium, thick, solid, globular, yellowish brown to brown, with obtuse apex and dome-shaped spire; 5 rapidly increasing and convex whorls separated by deep suture; body whorl expanded, slightly shouldered, slightly descending in front; periphery weakly angulate in front of aperture, becoming round before peristome. Surface with thick growth lines, and fine spiral ribs. Aperture roundly lunate, white inside, with curved margin. Peristome white, expanded, reflected, thickened and glossy; columellar margin expanded. Upper lip decline quickly; inner lip thickly callous. Basal lip curved, forming a smooth junction with oblique columellar lip. Umbilicus narrow, more than two-third of its area covered by reflected columellar lip. Hump beside umbilicus present. Color pattern of 3–5 reddish brown spiral bands on upper surface and numerous wavy, reddish brown spiral bands of various thickness bands on base; subperipheral and subsutural bands much wider (Fig. 3C).

Penis short, swollen, with a rounded bulge in correspondence of verge. Epiphallus swollen, with short and thin penis retractor muscle. Flagellum thickened, tapering distally. Vas deferens long and thin. Vagina short and swollen. Bursa copulatrix oval with long and thin pedunculus, expanded at basal half its length. Verge long, bluntly conic, with widely-spaced transverse wrinkles basally, dense and weak longitudinal grooves apically. Inner penial wall supporting several weak and dense pilasters: proximally transverse surrounding verge, distally longitudinal (Fig. 4C).

##### Distribution.

Known only from Guizhou, China (Fig. 1).

##### Ecology.

This species inhabits limestone forest. The animals appeared sensitive to environmental condition and can not be observed in farmland. Animals copulate during April to August (May and June mostly), lay eggs in September-October which hatch in 30 to 40 days (Zhang 2008).

##### Comparative remarks.

This species is characterized in having a globular and solid shell, the swelling and gibbous last whorl, a roundly lunate aperture, an almost covered umbilicus, both transverse and longitudinal pilasters on inner penial wall, and a bluntly conic verge with transverse and longitudinal wrinkles. Shell size varied between the two sampled populations (Table 4), but the phylogeny and genetic distances agreed that they are the same species. Comparing with other species, the distinct monophyly on the phylogenetic tree, and sufficient genetic and morphological differences provide enough evidences of species separation. Hence, this taxon is raised to species rank.

#### Camaena connectens

Taxon classificationAnimaliaStylommatophoraCamaenidae

Dautzenberg & Fischer, 1906

Figs 3D , 4D , Table 4

Camaena cicatricosa var. connectens Dautzenberg and Fischer, 1906: 356.
Camaena cicatricosa connectens , Schileyko 2011.

##### Type locality.

Vietnam, Ha-Giang

##### Material examined.

Type material. Syntype. MNHN-IM 2000-2020.

Additional material see Table 4.

##### Diagnosis.

Shell sinistral, large, thick, solid, depressed-globular, yellowish brown to brown, with obtuse apex and low dome-shaped spire; 5 1/2 rapidly increasing and slightly convex whorls, separated by shallow suture; body whorl expanded, weakly shouldered, slightly descending in front; periphery bluntly angulate in front of aperture, becoming round behind peristome. Surface with rough growth lines, spiral ribs, and apparent malleation. Aperture roundly lunate, white inside, with curved margin. Peristome white, expanded, reflected, thickened and glossy; columellar margin expanded. Inner lip thickly callous; basal lip curved, forming a smooth junction with oblique columellar lip. Umbilicus narrow, more than two-third of its area covered by reflected columellar lip. Hump beside umbilicus present. Color pattern of a few faint, wavy, reddish brown spiral bands of various thickness bands; subperipheral and subsutural bands much wider (Fig. 3D).

Penis swollen, slightly tapering distally, with a rounded bulge in correspondence of verge. Epiphallus thick, with long and thin penis retractor muscle. Flagellum long, thick basally, tapering distally. Vas deferens short and thin. Vagina short and swollen. Bursa copulatrix elongated-oval with long and thin pedunculus, expanded at basal half. Verge bluntly conic, with dense, deep longitudinal grooves. Inner penial wall supporting proximally transverse, short, weak, narrowly-spaced wrinkles surrounding verge, and distally longitudinal, prominent and widely spaced pilasters (Fig. 4D).

##### Distribution.

This species has previously been recorded from Ha Giang in northern Vietnam only. In addition, it is now recorded from Tianbao, Malipo, southeastern Yunnan, China, approximately 20 km northwest of Ha Giang (Fig. 1).

##### Ecology.

This species inhabits humid limestone forest and can not be found in farmland.

##### Comparative remarks.

*Camaena connectens* can be distinguished from other sinistral *Camaena* species by having a rougher surface, an almost covered umbilicus, fewer and faint spiral bands, a hump beside umbilicus, both transverse and longitudinal pilasters on inner penial wall, and a bluntly conic verge with longitudinal grooves. *Camaena hahni broti* (Dautzenberg & d’Hamonville, 1887) resemble *Camaena connectens* in having a sinistral shell with rough surface, but the former has a nearly opened umbilicus, and carinate periphery. *Camaena connectens* differs from *Camaena cicatricosa* in having a larger shell, a narrower umbilicus, a hump beside umbilicus, and both transverse and longitudinal wrinkles on inner penial wall. This species can be distinguished from *Camaena obtecta* by having a larger shell, a wider umbilicus, a curved basal lip, and both transverse and longitudinal wrinkles on inner penial wall. This taxon is raised to species rank because of the well-differentiation of molecular and morphological characters.

#### Camaena poyuensis

Taxon classificationAnimaliaStylommatophoraCamaenidae

Zhou, Wang & Ding sp. n.

http://zoobank.org/78C95D9C-A54E-484B-889F-8640CD79DE11

**Figure 5.**
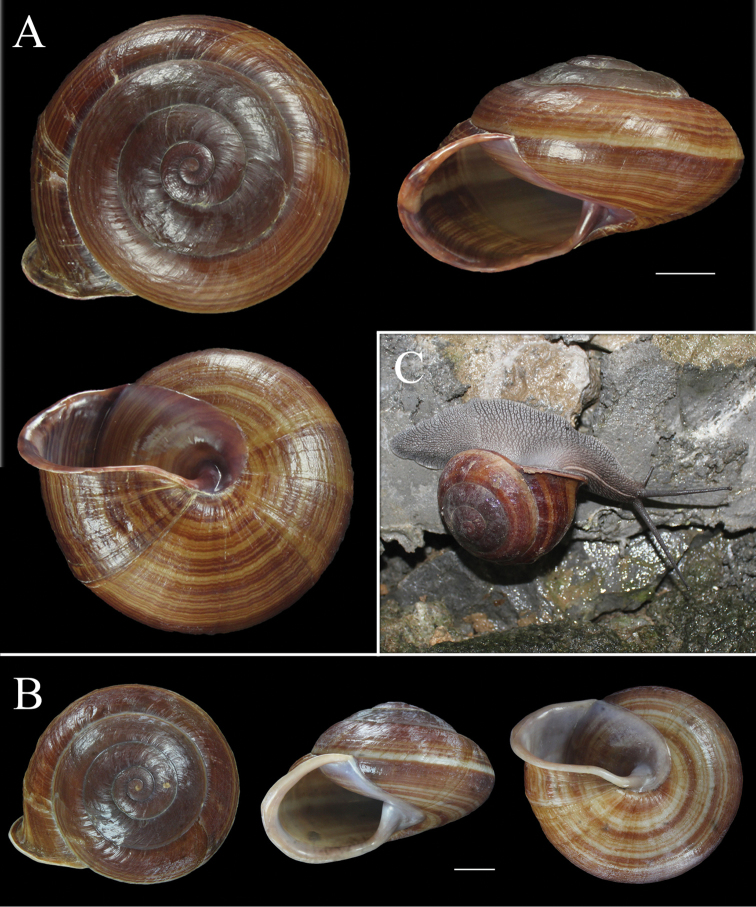
Shell of *Camaena poyuensis* sp. n. **A** Holotype, FJIQBC 18484, Poyue, Bama, Guangxi, China **B** Paratype, FJIQBC18489, from type locality **C** Life photograph of paratype, FJIQBC 18485, from type locality. Scale: 10 mm.

Figs 4E , 5 , Table 4

##### Material examined.

Holotype. FJIQBC 18484, specimen preserved in ethanol, China, Guangxi Zhuang Autonomous Region, Hechi City, Bama County, Poyue town, 24°17'30"N; 107°05'32"E (Fig. 1); limestone mountain, coll. WC Zhou, May 25, 2014.

Paratypes. 19 specimens with the same data as holotype but with the following specimen codes: 4 in ethanol (FJIQBC 18485–18488), 2 adults; 15 empty shells (FJIQBC 18489–18503), 9 adults.

Measurements of shells see Table 4.

##### Diagnosis.

Shell sinistral, large, thick, discoidal, with obtuse apex and low dome-shaped spire; 5 1/2 rapidly increasing and slightly convex whorls separated by deep suture; body whorl expanded; peripheral angle blunt. Surface with thick growth lines, and fine spiral ribs. Aperture lunate, angulated by peripheral carina. Peristome expanded, reflected, thickened and glossy. Inner lip thin, forming a smooth, semi-translucent, and purplish callus. Basal lip and columellar lip straight, with obtuse angle at junction. Umbilicus covered completely by reflected columellar lip. Color pattern of several wavy, reddish brown spiral bands of various thickness, peripheral and subsutural bands much wider; spire dark brown. Peristome and callus tinted purplish (Fig. 5A), fading to red-dotted pink on dead-collected shells (Fig. 5B).

Animal light brown, tentacles dark brown, distinct yellowish line, running from the head between tentacles to the collar near the peristome (Fig. 5C). Penis swollen, tapering distally, with a rounded bulge in correspondence of verge. Epiphallus thick, with short, thin and wide penis retractor muscle. Flagellum slender, tapering distally. Vas deferens long and thin. Vagina long and thin, thickened proximally. Bursa copulatrix head oval with long and thin pedunculus, expanded at base. Verge short, conic, with six longitudinal grooves extending from verge base to about three quarters of its length and narrowly-spaced transverse wrinkles. Inner penial wall supporting several pilasters: proximally transverse, weak and dense surrounding verge, distally longitudinal, prominent and widely-spaced (Fig. 4E).

##### Etymology.

For the type locality, adjective of feminine gender.

##### Distribution.

This species is known from the type locality only.

##### Ecology.

The new species habits in a well-preserved subtropical evergreen broadleaved forest, and is not common. The animal was not found in farmland adjacent to the forest.

##### Comparative remarks.

Diagnostic comparisons of morphological characters of the new species and the other four *Camaena* were summarized in Table 5. The new species and *Camaena cicatricosa* are sister taxa (Fig. 2) and similar in shell shape, color pattern and absence of a hump beside umbilicus. The shell of *Camaena cicatricosa* differs from the new species by having a smaller shell, higher spire, a half opened umbilicus, a more dilated columellar lip, peristome white, curved basal lips. Among the sinistral species of the subgenus Camaena (Camaena), only *Camaena obtecta* (Fischer, 1898) and the new species have a totally covered umbilicus and, hence, can be distinguished from the others. *Camaena obtecta* shows thick umbilical callus, a hump beside the margin of the callus, white peristome, and a thicker shell and a higher spire than the new species.

**Table 5.** Diagnostic comparisons of morphological characters of five sinistral *Camaena* from China.

Character	*Camaena cicatricosa*	*Camaena obtecta*	*Camaena inflata*	*Camaena connectens*	*Camaena poyuensis* sp. n.
SW (mm)	33.8–48.7	53.2–61.9	38.4–56.7	48.2–55.3	52.5–58.7
Umbilicus	half covered	completely covered	almost covered	almost covered	completely covered
Shell shape	depressed-globose	depressed-globose	globose	depressed-globose	depressed-globose
Shell thickness	thin	thick	thick	thick	thin
Basal lip	curved	straight	curved	curved	straight
Hump beside umbilicus	absent	humped	humped	humped	absent
Pilaster on inner penial wall	longitudinal, narrowly-spaced	transverse, narrowly-spaced	proximal: transverse, narrowly-spaced distal: longitudinal, narrowly-spaced	proximal: transverse, widely-spaced wrinkles distal: longitudinal, widely-spaced	proximal: transverse, narrowly-spaced distal: longitudinal, widely-spaced
Verge	long conic	conic	bluntly long conic	bluntly conic	short conic
Verge surface	longitudinal, dense, weak wrinkles	irregularly curly, weak wrinkles	Proximal: transverse, deep wrinkles distal: longitudinal, dense, shallow wrinkles	longitudinal, dense, deep grooves	longitudinal, deep grooves

The morphology of the reproductive system of *Camaena poyuensis* is similar to *Camaena cicatricosa*, but differs in the following characters: The pedunculus of the bursa copulatrix is longer, more than twice as long as the vagina (it is about as long as the vagina in *Camaena cicatricosa*). The epiphallus and penis are thicker than in *Camaena cicatricosa*, and the penis has a visible projection. *Camaena poyuensis* sp. n. has shorter verge with both transverse and longitudinal grooves, and transverse pilasters in proximal part of penis. The verge of *Camaena obtecta* is similar to that of *Camaena poyuensis* sp. n. in shape, but its surface is covered throughout with irregular, fine and curly wrinkles. Only transverse pilasters are present in the penis of *Camaena obtecta*, whereas both the transverse and longitudinal pilasters are seen in the new species.

## Discussion

The systematics of four of the five subspecies of *Camaena cicatricosa* has been revised in this study. The genetic distances of the COI barcoding region between *Camaena cicatricosa*, *Camaena inflata*, *Camaena obtecta*, *Camaena connectens* and the new species agree with the interspecific genetic distances of other camaenid groups, such as, for example, the Australian camaenid *Kimberleytrachia* (0.055–0.161, Criscione and Köhler, 2014), the Japanese camaenid *Luchuhadra* (0.003–0.205, Kameda et al. 2007) and the Taiwanese camaenid *Satsuma* (0.006–0.150, Wu et al. 2008). In addition to genetic distance, phylogenetic topography and morphological differences also support that they are distinct species. Nomenclatural acts were applied to these taxa, which include raises of taxonomic rank of three species and description of a new species.

Live specimens of two Chinese sinistral *Camaena* were not collected in the present study: *Camaena cicatricosa ducalis* and *Camaena seraphinica*. Their systematic position are not revised and remained as their current taxonomic ranks. *Camaena cicatricosa ducalis* was named based on a single specimen collected from Kouy-Yang-Fou (nowadays Guiyang), Guizhou. No further specimens were confirmedly recorded since its publication. The present authors and Prof. Tai-Chang Luo (a malacologist based on Guizhou Normal University, Guiyang, personal communication) have spent decades in biodiversity survey in Guizhou, but no shell fullfil the original descriptions were collected (Luo et al. 2003). This taxon is characterized in its large shell width of 74 mm (Ancey 1885). The shell width of known species of sinistral Camaena (Camaena) are hardly larger than 62 mm. Owing to its rarity and unusual size, this subspecies is possibly merely a gigantic variation of one of the *Camaena* species or extinct. *Camaena seraphinica* (Fig. 3E) differs from the *Camaena cicatricosa* species complex by having a totally open umbilicus, a strongly descending aperture, a non-malleated surface, and a white shell background with few wide bands.

The molecular data also confirms the widely distributed *Camaena cicatricosa* a well-delineated species by including samples from subtropical lowland and hill region of Guangdong and eastern Guangxi Province. An extreme example is that individuals from Shantou and Nanning, populations that are about 1000 km apart (Fig. 1), shared the same sequences of the three genes. The large distributional range of this species and short genetic distances among populations are probably due to its adaptation to mankind-disturbed environments, such as farmland and forest ecotone. They have the higher probability to be passively transported large distances and to establish a new population, through human activities (Aubry et al. 2006, Guiller and Madec 2010). Besides, species of *Camaena* demonstrate different life history strategies in preliminary field and laboratory observations. *Camaena cicatricosa* laid more but smaller eggs (10–25 eggs each clutch, 0.2–0.25 g each egg) than *Camaena inflata* (5–9 eggs each clutch, 0.38 g each egg) (Zhang 2008, Xiao 1989). The former also has shorter gestation period (5–36 days) between the last copulation and the first egg-laying than the latter (2 months). Organisms having higher fecundity and, hence, higher abundance tend to be more competitive species (Stearns 1992). This may partly explain the dominance of *Camaena cicatricosa* in these areas. A thorough sampling for a phylogeographic analysis and comparative studies of life history of *Camaena cicatricosa* and its allies can provide a considerable insight into the evolutionary processes of *Camaena cicatricosa*.

Most of Camaena (Camaena) are distributed in the area of southwestern China and northern Vietnam, where is located at northern part of the Indo-Burma Hotspot and at transition to the Mountains of Southwest China Hotspot (Myers et al. 2000). Three of the species, *Camaena obtecta*, *Camaena connectens* and *Camaena poyuensis* sp. n., studied in the present research are distributed in the Indo-Burma Hotspot. The complex topography, varied physical conditions and a wide diversity of ecosystems in these mountainous areas have likely resulted in allopatric and sympatric speciation (Harl et al 2014, Criscione and Köhler 2016) and hence, a high biodiversity of land snails is expected (Yen 1939, Schileyko 2011).

The present molecular data set using three genetic markers supports the previous separations of *Camaena cicatricosa* based exclusively on shell morphology. However, more work is needed to sort out some systematic issues: (1) the taxonomy and phylogenetic relationship of all of the *Camaena* species, especially those inhabit around the border between Vietnam and China (2) the mechanism of speciation of *Camaena* in this area (3) the phylogeography of *Camaena cicatricosa*, the most widely distributed species in China.

## Supplementary Material

XML Treatment for Camaena

XML Treatment for Camaena cicatricosa

XML Treatment for Camaena obtecta

XML Treatment for Camaena inflata

XML Treatment for Camaena connectens

XML Treatment for Camaena poyuensis
